# Relationships between *UBE3A* and *SNORD116* expression and features of autism in chromosome 15 imprinting disorders

**DOI:** 10.1038/s41398-020-01034-7

**Published:** 2020-10-29

**Authors:** Emma K. Baker, Merlin G. Butler, Samantha N. Hartin, Ling Ling, Minh Bui, David Francis, Carolyn Rogers, Michael J. Field, Jennie Slee, Dinusha Gamage, David J. Amor, David E. Godler

**Affiliations:** 1 Diagnosis and Development, Murdoch Children’s Research Institute, Royal Children’s Hospital, Melbourne, Victoria Australia; 2 Faculty of Medicine, Dentistry and Health Sciences, Department of Paediatrics, University of Melbourne, Parkville, Victoria Australia; 3 School of Psychology and Public Health, La Trobe University, Melbourne, Victoria Australia; 4 Department of Psychiatry, Behavioral Sciences and Pediatrics, University of Kansas Medical Centre, Kansas City, Kansas USA; 5 Centre for Epidemiology and Biostatistics, Melbourne School of Population and Global Health, University of Melbourne, Melbourne, Victoria Australia; 6 Victorian Clinical Genetics Services and Murdoch Children’s Research Institute, Royal Children’s Hospital, Melbourne, Victoria Australia; 7 Genetics of Learning Disability Service, Hunter Genetics, Waratah, New South Wales Australia; 8 Department of Health, Government of Western Australia, Genetic Services of Western Australia, Perth, Western Australia Australia; 9 Neurodisability and Rehabilitation, Murdoch Children’s Research Institute, Royal Children’s Hospital, Melbourne, Victoria Australia

**Keywords:** Human behaviour, Biomarkers, Clinical genetics

## Abstract

Chromosome 15 (C15) imprinting disorders including Prader–Willi (PWS), Angelman (AS) and chromosome 15 duplication (Dup15q) syndromes are severe neurodevelopmental disorders caused by abnormal expression of genes from the 15q11–q13 region, associated with abnormal DNA methylation and/or copy number changes. This study compared changes in mRNA levels of *UBE3A* and *SNORD116* located within the 15q11–q13 region between these disorders and their subtypes and related these to the clinical phenotypes. The study cohort included 58 participants affected with a C15 imprinting disorder (PWS = 27, AS = 21, Dup15q = 10) and 20 typically developing controls. Semi-quantitative analysis of mRNA from peripheral blood mononuclear cells (PBMCs) was performed using reverse transcription droplet digital polymerase chain reaction (PCR) for *UBE3A* and *SNORD116* normalised to a panel of internal control genes determined using the geNorm approach. Participants completed an intellectual/developmental functioning assessment and the Autism Diagnostic Observation Schedule-2nd Edition. The Dup15q group was the only condition with significantly increased *UBE3A* mRNA levels when compared to the control group (*p* < 0.001). Both the AS and Dup15q groups also had significantly elevated *SNORD116* mRNA levels compared to controls (AS: *p* < 0.0001; Dup15q: *p* = 0.002). Both *UBE3A* and *SNORD116* mRNA levels were positively correlated with all developmental functioning scores in the deletion AS group (*p* < 0.001), and autism features (*p* < 0.001) in the non-deletion PWS group. The findings suggest presence of novel interactions between expression of *UBE3A* and *SNORD116* in PBMCs and brain specific processes underlying motor and language impairments and autism features in these disorders.

## Introduction

Angelman syndrome (AS), Prader–Willi syndrome (PWS) and chromosome 15 duplication syndrome (Dup15q) are neurodevelopmental disorders that are associated with varying degrees of intellectual disability (ID) and social communication deficits^1,2^, and arise from different deletions or duplications at the 15q11–q13 imprinted region^3^.

PWS was the first example of genomic imprinting identified in humans^4^. Cardinal features include a poor suck with failure to thrive, infantile hypotonia and hypogonadism. Food seeking and hyperphagia emerges at approximately 6 years of age, leading to morbidity if not externally controlled. Mild ID (mean full scale IQ [FSIQ] between 55 and 69) is typical, frequently accompanied by compulsions, tantrums and skin picking^5^. AS is characterised by microcephaly, gait ataxia, seizures, ID, and absence of speech^6^. Dup15q is associated with variable cognitive impairment and motor delays. An overlapping feature between AS and Dup15q is the presence of seizures^3^.

DNA methylation and/or copy number changes on chromosome 15 are thought to cause PWS and AS specific phenotypes^3,7^. Loss of paternal gene expression from the chromosome 15q11–q13 region is the primary cause of PWS^7^, while the absence of the maternal gene expression in the same region is the primary cause of AS^3^. For PWS the lack of expression of key genes result from: (i) two deletion subtypes (typical—type I and type II deletions; and atypical smaller or larger 15q deletions) in ~60% of cases; (ii) three maternal disomy subtypes in ~35% of cases; and (ii) two imprinting centre defects (ICD; epimutation and microdeletion) in ~5% of cases^5,8^. Similarly, deletions from the maternally contributed chromosome 15 are the most common cause of AS (~70% of cases). Paternal uniparental disomy (patUPD) occurs in approximately 8% of AS cases and ICD in approximately 7% of cases^3^. Approximately, 10% of AS cases result from a mutation in the ubiquitin-protein ligase E3A gene (*UBE3A*). Both PWS and AS have a frequency of approximately 1 in 15,000 births^9^.

Dup15q syndrome results from duplications or triplications of the PWS/AS imprinted 15q11–q13 region. Triplication typically arises through the presence of a supernumerary chromosome (isodicentric 15 [idic15]), while the duplication is caused by interstitial tandem duplication (int dup[15]). Hereafter, we use Dup15q to encompass these subtypes, unless otherwise stated. In maternal Dup15q, autism features are more common and severe, as compared to AS and PWS, with severity directly proportional to the number of maternal copies present^9^. In contrast, paternal Dup15q has a less severe phenotype than maternal Dup15q^10^. Despite Dup15q being a cause of autism spectrum disorder (ASD), reported in 1–3% of ASD cases^9^, prevalence in the general population has not been well established, with one study reporting 1 in 14,000 in the general population^11^.

While some genotype–phenotype correlations have emerged in each of the syndromes, primarily around the different molecular classes, there is a need for peripheral tissue biomarkers in humans as the phenotypes are highly variable in each disorder, and their specific subtypes do not fully explain this variability^12^. For PWS, those with the typical 15q11–q13 deletions have been reported to have lower Verbal IQ (VIQ) scores than those with matUPD^13^. In addition, PWS individuals with the larger typical 15q11–q13 type I deletion involving chromosome 15 breakpoints BP1 and BP3 have been reported to have more behavioural problems, specifically self-injury and compulsions compared to those having the smaller typical 15q11–q13 type II deletion involving breakpoints BP2 and BP3^14^. The larger type I deletion encompasses four extra genes (i.e., *NIPA2*, *NIPA1*, *GCP5*, and *CYFIP1*), which may account for the additional clinical findings.

For AS, mouse models have been used to implicate loss of *UBE3A* expression in the brain as the primary cause of specific deficits in AS^15^. However, few studies have been performed in human peripheral tissues^16,17^. *UBE3A* is a key gene in neurodevelopment and is thought to be imprinted only in neurons, where it is expressed from the maternal allele in humans and mice^15^. Interestingly in other cell types *UBE3A* has bi-allelic expression^18^ and is thought to be consistently expressed from both alleles in different peripheral tissues^15^. The silencing of *UBE3A* on the paternal allele in neurons is thought to be regulated by a paternally expressed antisense transcript of *UBE3A*. This antisense transcript is part of the 3′end of *SNRPN–SNURF* transcript that comprises multiple small nuclear RNAs (snoRNAs)^19^. SnoRNA C/D box cluster 116 (*SNORD116*) is one of the snoRNAs that serves as a precursor to the antisense *UBE3A*, and through this process may regulate *UBE3A* silencing on the paternal allele^16^. Importantly, in PWS *SNORD116* has been reported to be completely silenced, in neurons as well as other cell types in peripheral tissues, and this may contribute to phenotype severity^17^.

This study, for the first time, examines *UBE3A* and *SNORD116* mRNA levels (controlled for the allele copy number and subtype) in peripheral blood mononuclear cells (PBMCs) using a highly sensitive and quantitative droplet digital PCR (ddPCR) method developed for analysis of gene expression. Expression changes for these genes in PBMCs are investigated between the chromosome 15 imprinting disorders, the different subtypes and typically developing controls. For AS and PWS subtypes genotype–phenotype studies are also described, with the focus on relationships between the *UBE3A* and *SNORD116* mRNA levels in PBMCs and brain specific phenotypes including formal assessments targeting behavioural features and intellectual functioning. It was hypothesised that expression of these genes in PBMCs reflects immune processes in the brain related to gene expression in microglial cells (of the same cellular lineage) that support neuronal processes in the brain related to intellectual/developmental functioning and autism features in these disorders^20^.

## Subjects and methods

This study comprised 58 individuals with a chromosome 15 imprinting disorder aged between 1 and 45 years. Twenty-seven individuals with PWS, 21 individuals with AS and 10 individuals with Dup15q were included in the study. The molecular classes for each syndrome are presented in the supplementary material (Table S1). Samples from 20 de-identified controls, aged 8–52 years, from our earlier studies^21,22^ were included as reference data for gene expression comparisons.

Participants were recruited through Victorian Clinical Genetics Services; Hunter Genetics; the PWS Clinic at the Royal Children’s Hospital, Melbourne; Genetic Services of Western Australia; and various support organisations including the Foundation for Angelman Syndrome Therapeutics, the PWS Clinic at the Royal Children’s Hospital, Melbourne; the Prader–Willi Association of Australia. Inclusion criteria were that verbal participants be able to speak English and non-verbal participants to be exposed to English within the home. Exclusion criteria were having another genetic condition of known clinical significance, or having any significant medical condition (e.g., stroke and head trauma).

For all participants their diagnosis was confirmed using DNA methylation analysis, methylation-specific multiple ligation-dependent probe amplification and/or microarray testing for copy number changes within the 15q11–q13 region, as per standard diagnostic testing protocol^23^.

### Sample processing

At the time of assessment, 5 ml of venous blood were collected in ethylenediaminetetraacetic acid tubes, followed by PBMC isolation using Ficoll gradient separation, as previously described^24^. RNA was extracted from 1 to 4 million viable PBMCs using RNeasy kit as per manufacturer’s instructions (Qiagen, Germany). RNA samples were diluted between approximately 10 and 20 µl/ng depending on the concentration, resulting in an average input of 100 ng per reverse-transcription reaction. Reverse transcription was performed using the High-Capacity cDNA Reverse Transcription Kit as per manufacturer’s instructions (Thermos-Fisher, Global).

### Gene expression analysis utilising ddPCR

To establish the optimal reference genes for our samples, eight assays (*EIF4A2*, *RPL13A*, *SDHA*, *TOP1*, *YWHAZ*, *APT5B* and *GAPDH*) were used from the geNorm reference 12 gene kit, as per manufacturer’s instructions (PrimerDesign, Ltd., Camberley, United Kingdom). For each assay, 1.1 µl of the primer/probe mix was added to 1× ddPCR SuperMix for probes (no dUTPs) (Biorad, Global) and RNAse/DNAse free water, and then aliquoted into 96-well PCR plates. One to two microlitre of cDNA was then added to the reaction mix.

For target genes, *SNORD116* used the forward (5′-CAGGAAAGATCAAAACGATGCA-3′) and reverse (5′-TCCAAAGGAGGCAGTTGGAT-3′) primers with a HEX labelled probe (5′- TGCAAGTGTGATTGGTCCAGATAGCTGC-3′). This target region was referred to as *SNURF–SNRPN* exons 57–58, in a previous study^16^. The *UBE3A* assay was a premade TaqMan Assay (ID: Hs00166580_m1) with specific primer sequences not made available by the manufacturer (Thermo Fisher Scientific, MN, USA). The assay targets an amplicon with Chr.15: 25337234–25439086 coordinates [on Build GRCh38] present in most *UBE3A* transcripts, utilising a FAM-labelled probe. Both *UBE3A* and *SNORD116* assays utilised a Deep Hole Quencher and were duplexed. Briefly, each 20 μL reaction mixture contained 2.0 μl cDNA, 1× ddPCR SuperMix for probes, no dUTPs (Bio-Rad Laboratories; Hercules, California), 125 nM *SNRPN* forward primer, 125 nM *SNRPN* reverse primer, 500 nM *SNRPN* HEX tagged probe, 1× *UBE3A* FAM-tagged TaqMan assay, and RNAse/DNAse free water. Prepared reactions were run on a Bio-Rad QX200 system (Bio-Rad, Hercules, California). Data were analysed using the QuantaSoft Analysis Pro software, as per manufacturer’s instructions (Bio-Rad, Hercules, California), with data collected from two different channels specific for HEX and FAM signals (with no confirmed signal overlap).

### Intellectual functioning

All individuals with AS were assessed with the Mullen Scales of Early Learning (MSEL)^25^, a developmental assessment that assessed the domains of visual reception, fine motor, receptive language, and expressive language. Developmental assessments are commonly used for individuals with AS and Dup15q, given the significant developmental delay that is commonly observed in these individuals and the subsequent propensity to have scores that fall at the “floor” on standardised age-appropriate assessments. The MSEL provides age equivalent scores in months for the domains of visual reception, fine motor, receptive language and expressive language. All PWS participants completed an age appropriate standardised assessment. Standardised intellectual functioning assessments included the MSEL (children aged under 3 years), the Wechsler Preschool and Primary Scale of Intelligence-3rd Edition (WPPSI-III; children aged 3–6 years, 11 months)^26^, the Wechsler Intelligence Scale for Children-4th Edition Australian (WISC-IV; Australian children aged 7–16 years, 11 months)^27^ or the Wechsler Adult Intelligence Scale-4th Edition Australian edition (WAIS-IV; individuals aged 17+ years)^28^. The following intellectual functioning variables were included in the analyses: VIQ, performance IQ (PIQ) and FSIQ.

### Autism features

The Autism Diagnostic Observation Schedule-2nd Edition (ADOS-2)^29^ was used to assess social communication skills and the presence of repetitive and restricted behaviours. The ADOS-2 was used in all children who were cruising/walking and had a mental age of 12 months or greater. The ADOS-2 provides an overall calibrated severity score (CSS), in addition to CSS for the social affect (SA CSS) and repetitive and restricted behaviours (RRB CSS) domain. All assessments were completed by the first author, a certified ADOS-2 trainer in Australia, who regularly engages in reliability coding meetings to maintain research reliability of >80%.

### Procedures

Participants attended an appointment at a clinic or within their own home for the developmental/intellectual functioning assessment and the ADOS-2. Venous blood was collected at the cessation of the clinical appointment. All procedures were approved by The Royal Children’s Hospital Human Research Ethics Committee (HREC #33066). All parents/caregivers provided written informed consent, and those aged 18 years and above, who were deemed cognitively able, also provided written consent.

### Data analysis

**Fig. 1:**
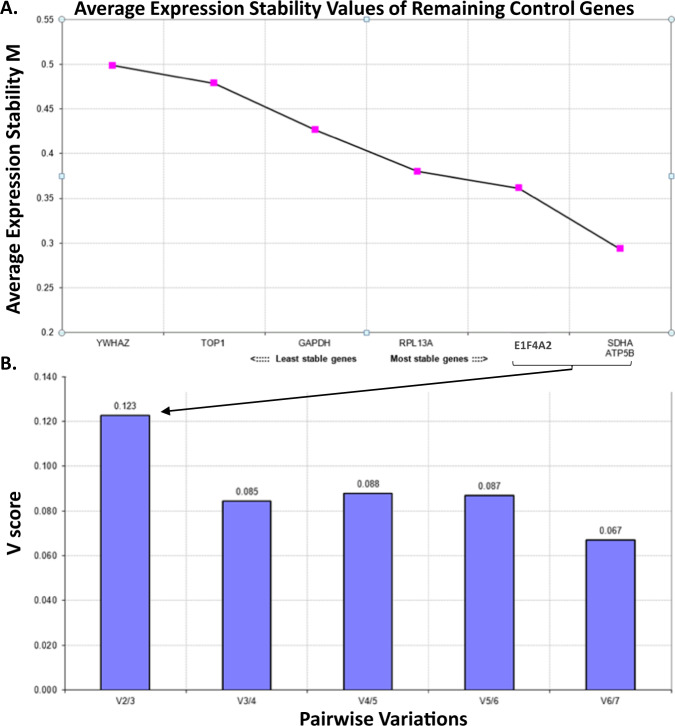
Selection of most stably expressed internal control genes in PWS, AS and control groups using the geNorm approach. Determining stability of expression for 7 internal control genes using the geNorm approach in peripheral blood mononuclear cells (PBMCs) of 44 individuals affected with PWS (*n* = 21) and typically developing controls (*n* = 23). **A** Average expression stability *M* values, with least to most stable ordered in the left to right direction on the *x*-axis. *SDHA*, *ATP5N* and *E1F4A2* were the most stably expressed genes from the panel tested. **B** Variation in average gene expression stability with sequential addition of each internal control gene to the equation (for calculation of the *V* score normalisation factor). In **A**, the least stably expressed genes are shown on the left side of the *x*-axis and the most stably expressed genes or combinations of genes are shown on the right side (i.e., *ATP5B* and *SDHA*). *Note*: All internal control primer/probe mixes were obtained from PrimerDesign (PerfectProbe ge-PP-12-hu kit) and used at a concentration of 2 μM.

*UBE3A* and *SNORD116* mRNA copy numbers were normalised to copy numbers from a stably expressed set of internal control genes determined using the geNorm approach^30^ on the same set of cDNA samples from PWS (*n* = 21) and control (*n* = 23) cohorts (Fig. 1). Given the sample size for each group was small, the summary statistics were presented by median and interquartile range, and the nonparametric Kruskal–Wallis test or Mann-Whitney test were used for comparisons of the groups. For binary data, such as sex, the percentage was given and Fisher’s exact test was used for comparison. The robust regression, using M-estimator and robust standard errors, was used to assess the relationship between each phenotypic measure (intellectual functioning and ADOS scores) and *UBE3A* and *SNORD116* mRNA levels. Adjustments for confounders was undertaken where appropriate, especially for age, where the youngest participant was 0.8 years and the oldest was 45.6 years (e.g., age adjusted for MSEL equivalent scores in AS or for ADOS in Dup 15q group, and age and FSIQ for ADOS in PWS). The Bonferroni correction method was used to correct for multiple testing. All analyses were conducted using the software STATA (http://www.stata.com).

## Results

### Technical validation of ddPCR assays

Nine ddPCR assays were validated including seven from an internal control gene panel (Supplemental Fig. 1) and two target genes, *UBE3A* and *SNORD116* (Supplemental Fig. 2). The validation experiments involved establishing positive call threshold and dynamic linear range (DLR) for each assay on serially diluted RNA reference sample, with RNA input ranging between 170.4 and 1.33 ng. For all assays the positive call thresholds were set on the ddPCR 2-D plots using no template controls (no RNA input—1st column of each 2-D plot included in every run) at the amplitude of the droplet/s with the highest amplitude unit value, representing non-specific signal.

For the *GAPDH* assay that showed multiple distinct droplet population above the non-specific signal amplitude in the no-template control sample, we trialled two different thresholds. The second threshold was set just below the most prominent positive droplet population. Supplemental Fig. 1D and 1E demonstrate that the correlation between expected and observed RNA input for the *GAPDH* essay are not significantly different between the no-template control-based threshold and the higher amplitude threshold set below the main positive droplet population. All of the internal control assays showed the DLR between 170.4 and 5.325 ng RNA input, with every serial dilution there was a proportional decrease in copy numbers detected. However, the target gene assays showed wider DLR, between 170.4 and 2.7 ng RNA input for *SNORD116*, and 170.4 and 1.33 ng RNA input for *UBE3A*. Subsequently, all RNA samples from chromosome 15 imprinting disorder cohorts and controls were diluted to equate to 10 ng input per cDNA synthesis reaction. This input overlapped with the approximate middle dilution for DLR of all internal control and target gene assays tested.

### Selection of most stably expressed internal control genes

The geNorm strategy was used to determine the minimum number of genes required to calculate a reliable normalisation factor for gene expression studies in PBMCs of 21 individuals with PWS and 23 typically developing controls (Fig. 1). Of the seven reference genes evaluated for different abundance and functional classes, the geometric mean of mRNA levels for *SDHA*, *ATP5B* and *EIF4A2* provided an accurate normalisation factor in the system tested. This is evident from the geNorm *V* score of 0.123, which is below the recommendation of the *V* score of 0.15 used to assess if expression of the selected genes are sufficiently stable. Moreover, inclusion of additional genes in the geometric average of the top three most stably expressed genes, did not significantly impact the *V* score for gene expression normalisation in the tested settings (Fig. 1B).

### Intergroup comparisons on clinical measures, *UBE3A* and *SNORD116* mRNA levels

The AS, PWS and Dup15q groups did not significantly differ on age at time of assessment (PWS: median (Md) = 7.15, interquartile range (IQR) = 12.31; AS: Md = 6.93, IQR = 16.03; Dup15q: Md = 6.38, IQR = 9.83; *p* = 0.672). Similarly, the proportion of males in each group did not significantly differ (PWS: 40.7%; AS: 52.4%; Dup15q: 60.0%; *p* = 0.520). Both the Dup15q and PWS groups had a significantly higher ADOS-2 CSS and SA CSS compared to the AS group (Table 1), but the three groups did not differ on RRB CSS. The PWS and Dup15q groups did not significantly differ on ADOS-2 scores. Descriptive intellectual functioning data are provided in Supplemental Table S2.

**Table 1:** Summary statistics and comparison between groups on ADOS-2 scores and *UBE3A* and *SNORD116* expression.

	PWS			AS			Dup15q						
	*n*	Md	IQR	*n*	Md	IQR	*n*	Md	IQR	*p*^123^	*p*^12^	*p*^13^	*p*^23^
ADOS CSS	25	6.0	5.0	20	3.5	2.0	8	7.0	1.0	**<0.001**	0.013*	0.127	<0.001*
SA CSS	25	5.0	4.0	20	3.0	2.0	8	8.0	3.0	**<0.001**	0.003*	**0.031**	<0.001*
RRB CSS	25	7.0	4.0	20	6.0	1.5	8	7.5	3.0	0.603	0.392	0.974	0.393
*UBE3A*	27	0.16	0.11	21	0.13	0.10	10	0.39	0.11	**<0.001**	0.247	<0.001*	<0.001*
*SNORD116*	–	–	–	21	0.04	0.02	10	0.04	0.01	–	–	–	0.852

*n* sample size, *Md* median, *IQR* interquartile range, *p value* (*p*) for comparison the difference between ^123^three subgroups, ^*12*^*PWS and AS* ^13^PWS and Dup15q and ^23^AS and Dup15q.

*p value remained <0.05 after Bonferroni correction.

*P* values in bold were statistically significant.

**Fig. 2:**
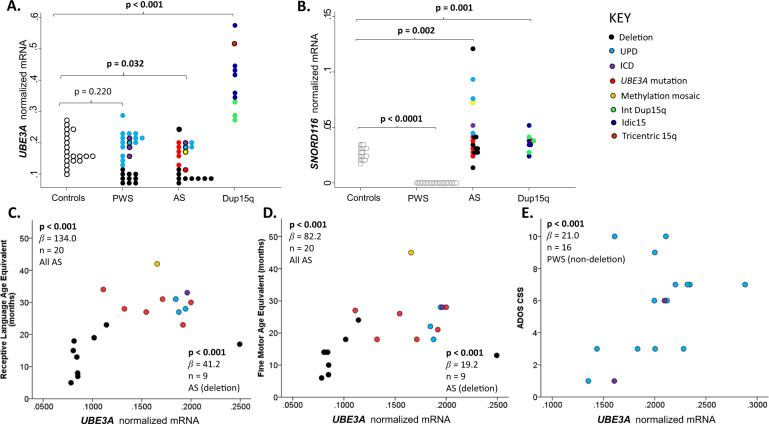
Intergroup comparisons and genotype–phenotype correlations for *UBE3A* and *SNORD116* mRNA levels in PBMCs. Intergroup comparisons between PWS, AS, Dup15q and control cohorts for **A** *UBE3A* and **B** *SNORD116* mRNA levels. Relationships between *UBE3A* mRNA levels in peripheral blood mononuclear cells (PBMCs) with **C** receptive language and **D** fine motor age equivalent scores in AS; **E** autism features assessed using ADOS CSS in PWS. Note: *UBE3A* mRNA levels were normalised to mRNA levels of *EIF4A2*, *SDHA*, and *ATP5B* (determined to be stably expressed using the geNorm approach in Fig. 1).

The Dup15q group had significantly elevated *UBE3A* mRNA levels compared to the PWS, AS and control groups (Table 1; Fig. 2A), while the AS, PWS and control groups did not significantly differ on *UBE3A* mRNA levels (Table 1; Fig. 2A). The AS and Dup15q groups did not significantly differ on *SNORD116* mRNA levels (Table 1). The normalised levels of *SNORD116* mRNA were significantly elevated in the AS and Dup15q groups as compared to the control group (Fig. 2A, B). In the Dup15q group the *UBE3A* median was more than threefold higher than the levels found in the AS group (Table 1) and more than twofold higher than controls (Fig. 2A). *SNORD116* mRNA levels showed the greatest variability in the AS group (range: 0.015–0.121 a.u). For the Dup15q group *UBE3A* mRNA levels (range: 0.268–0.574) were significantly more variable than those for *SNORD116* mRNA (range: 0.026–0.052). In contrast *SNORD116* mRNA was completely absent in the PWS group, but the levels of *UBE3A* mRNA were not significantly different between the PWS and control groups (Fig. 2A).

Comparison of deletion and non-deletion subtypes in the AS and PWS groups demonstrated that the deletion groups had significantly lower *UBE3A* mRNA levels compared to their non-deletion counterparts (AS: *p* = 0.002; PWS: *p* < 0.001; Supplement Table S3). However, *SNORD116* mRNA levels did not differ between the deletion and non-deletion subtypes in AS individuals (*p* = 0.197; Supplement Table S3). *UBE3A* mRNA levels in the methylation mosaic, ICD and UPD individuals were considerably higher (almost double) than those found in the deletion subtype (Fig. 2B) with the exception of one deletion individual who had the highest *UBE3A* mRNA level in the AS group.

### Relationships between mRNA and phenotypic measures

In the AS deletion subtype, both *UBE3A* and *SNORD116* mRNA levels were significantly associated with all age equivalent (months) scores derived from the MSEL (Table 2; Fig. 2C and 2D) and RRB CSS. Although higher *UBE3A* mRNA levels were significantly associated with higher expressive language age equivalent scores in the non-deletion AS subtype, this relationship did not survive adjustment for multiple comparisons. *SNORD116* expression was not significantly associated with any MSEL age equivalent or ADOS scores in the non-deletion subtype (Table 2). Analyses for the combined AS group showed significant associations between *UBE3A* expression and receptive and expressive language age equivalent scores (Table S4), primarily driven by the deletion subtype.

**Table 2:** Relationship between *UBE3A* and *SNORD116* expression and developmental functioning and autism features for deletion and non-deletion AS subtypes.

	Non-deletion (*n* = 11)				Deletion (*n* = 9)			
	*β*	s.e.	*p*	95% CI	*β*	s.e.	*p*	95% CI
*UBE3A*—predictor								
Visual reception+	−97.2	50.8	0.056	(−197, 2.36)	25.8	3.83	<0.001*	(18.3, 33.6)
Fine motor+	11.1	45.3	0.807	(−77.7, 99.8)	19.2	5.51	<0.001*	(8.42, 30.0)
Receptive language+	−36.2	30.6	0.236	(−96.2, 23.7)	41.2	6.85	<0.001*	(27.7, 54.6)
Expressive language+	40.4	20.3	**0.047**	(0.57, 80.2)	68.0	4.02	<0.001*	(60.1, 75.9)
ADOS CSS	17.0	11.8	0.151	(−6.19, 40.2)	−6.41	5.52	0.246	(−17.2, 4.42)
SA CSS	−6.11	12.2	0.616	(−30.0, 17.8)	−10.7	6.06	0.076	(−22.6, 1.13)
RRB CSS	12.7	19.9	0.544	(−33.3, 58.6)	−7.55	2.57	0.003*	(−12.6, -2.52)
*SNORD116*—predictor								
Visual reception+	−27.8	114	0.807	(−251, 195)	48.1	5.32	<0.001*	(37.7, 58.6)
Fine motor+	−16.7	78.3	0.831	(−170, 137)	42.6	10.1	<0.001*	(22.7, 62.4)
Receptive language+	25.5	65.3	0.696	(−103, 153)	78.1	9.24	<0.001*	(60.0, 96.2)
Expressive language+	31.8	132	0.809	(−227, 290)	117	8.52	<0.001*	(101, 134)
ADOS CSS	6.03	23.1	0.794	(−39.2, 51.3)	−9.60	8.81	0.276	(−26.9, 7.66)
SA CSS	−9.29	14.8	0.530	(−38.3, 19.7)	−18.8	10.7	0.078	(−39.7, 2.13)
RRB CSS	−0.09	19.1	0.996	(−37.5, 37.4)	−12.9	4.36	0.003*	(−21.4, -4.36)

*β* estimated regression coefficient, *s.e.* standard error, *p* value *p*, *CI* confidence interval, + adjusted for age in the deletion group.

*p Values remained <0.05 post Bonferroni correction.

*P* values in bold were statistically significant.

In the PWS non-deletion subtype, *UBE3A* mRNA levels were significantly negatively correlated with PIQ scores and FSIQ scores, though the latter did not survive adjustment for multiple comparisons (Table 3). *UBE3A* mRNA levels were also positively correlated with ADOS CSS (Fig. 2E) and SA CSS (Table 3), after adjustment for age at time of assessment and multiple comparisons (Table 3; Fig. 2E). In contrast, in the PWS deletion subtype, *UBE3A* mRNA levels were significantly negatively associated with SA CSS and ADOS CSSS, though the association with ADOS CSS did not survive Bonferroni adjustment. When the PWS group was analysed with all subtypes combined (Table S5), no significant associations were observed between ADOS scores and *UBE3A* mRNA levels.

**Table 3:** Relationship between *UBE3A* expression and intellectual functioning and autism features for deletion and non-deletion PWS subtypes.

	Non-deletion					Deletion				
	*n*	*β*	s.e.	*p*	95% CI	*n*	*β*	s.e.	*p*	95% CI
*UBE3A—predictor*										
VIQ	17	−92.4	95.0	0.331	(−279.0, 93.8)	10	−244.0	122.0	0.081	(−525.0, 37.3)
PIQ	17	−170.0	58.5	0.004*	(−285.0, -55.4)	10	−146.0	206.0	0.500	(−621.0, 330)
FSIQ	17	−148.0	69.2	**0.033**	(−2830, −12.2)	10	−95.3	218.0	0.674	(−599.0, 408)
ADOS CSS^a^	16	21.0	5.6	<0.001*	(10.1, 31.9)	9	−150.0	56.5	**0.037**	(−289.0, −12.1)
SA CSS^a^	16	11.4	3.5	0.001*	(4.5, 18.2)	9	−132.0	35.3	0.010*	(−219, −45.9)
RRB CSS^a^	16	25.8	15.6	0.098	(−4.8, 56.3)	9	−48.5	57.6	0.432	(−190.0, 92.5)

*β* estimated regression coefficient, *s.e.* standard error, *p value* *p*.

^a^Adjusted for age and FSIQ in non-deletion group.

*p values remained <0.05 post Bonferroni correction.

*P* values in bold were statistically significant.

In the Dup15q group *UBE3A* and *SNORD116* mRNA levels were not significantly associated with any of the ADOS scores (Table S6). Due to high variability in the intellectual functioning assessments used and small sample size, analyses were not conducted for intellectual/developmental functioning variables for the Dup15q group.

## Discussion

This study highlights the potential utility of gene expression in peripheral tissues as biomarkers for phenotypic severity in chromosome 15 imprinting disorders. While these syndromes are predominantly thought to be caused by abnormal functioning of neurons in the brain, the significant associations observed between *UBE3A* mRNA in peripheral blood and phenotypic variables suggest that changes in gene expression in PBMCs reflect brain specific processes indirectly measured through formal assessments of intellectual functioning and autism features. Specifically, this study demonstrated that increased *UBE3A* mRNA levels were associated with age equivalent scores derived from the MSEL in AS individuals with the deletion subtype and ADOS scores in PWS individuals, though the direction of association differed between the deletion and non-deletion PWS groups. No significant associations were observed between gene expression of *UBE3A* and *SNORD116* and autism features in the Dup15q group.

This study was also the first to utilise ddPCR, as a more precise and reproducible technique to quantify *UBE3A* and *SNORD116* mRNA levels from human peripheral tissues in chromosome 15 imprinting disorders. This may explain the associations between gene expression in blood and phenotype severity observed in this study, not previously examined. One of the strengths of the approach used is characterisation of the most stably expressed internal control genes using the geNorm approach in PBMCs of PWS and control groups in this study^30^. This approach is in line with the broad best practice recommendations for locus specific gene expression analyses^31^. However, we are not aware of previous studies examining gene expression in peripheral tissues of patients with chromosome 15 imprinting disorders that have tested a panel of genes (as in this study) to determine the optimal combination of most stably expressed genes, to be chosen as appropriate reference controls. This is a significant limitation of the earlier studies^16,32^ that needs to be taken into consideration, when interpreting and comparing results from this study with those conducted earlier.

Together, our approach addresses two potential technical limitations associated with previous gene expression studies in chromosome 15 imprinting disorders including: (i) use of techniques^15,16,32^ that are not as precise or quantitative as ddPCR for locus specific analysis^33^; (ii) target gene(s) normalised to a single internal control gene such as *β*-actin^16^, *β*-2 microglobulin^15^ or GAPDH^32^ that were not validated as part of gene expression stability studies, and may be themselves affected in the tested conditions. In this study, *SDHA*, *ATP5B* and *EIF4A2* were identified as the combination of reference genes most stably expressed in these settings, and were used to normalise expression of target genes, *UBE3A* and *SNORD116*. Future studies aiming to examine expression of other genes in these settings, now have evidence supporting use of the same normalisation strategy as described here for *UBE3A* and *SNORD116*.

### Dissociation between *UBE3A* and *SNORD 116* expression in PBMCs

The *SNORD116* mRNA levels showed the greatest variability in the AS group as compared to *UBE3A*. However, for *UBE3A* there was almost a clear split, with the deletion AS individuals showing lower mRNA levels than the non-deletion group, with the exception of one individual with AS and the deletion subtype, who had one of the highest levels of expression reported for all individuals examined. For *SNORD116* the *UBE3A* mutation group appeared to cluster with the deletion subtype. This suggests that there is a dissociation between *UBE3A* and *SNORD116* expression in AS, at least in peripheral blood; a similar phenomenon to what was observed in blood of PWS and AS patients previously^16^.

For the Dup15q group, the expression of *UBE3A* was the highest from all groups examined with significant variability observed. As expected, those with the int dup15 subtype had the lowest *UBE3A* mRNA levels within the Dup15q group. Moreover, the results for the combined Dup15q group were approximately four times those of controls. This is consistent with *UBE3A* not being imprinted in PBMCs, and further demonstrates the reliability of the ddPCR method developed and used to quantify mRNA levels in PBMCs in this study. However, this does not necessarily rule out that factors other than copy number changes impact mRNA levels in Dup15q individuals with the idic15 subtype, consistent with results previously observed in idic15^34^. In contrast, *SNORD116* mRNA levels were less variable within the Dup15q group, with expression being not significantly different from the AS group, but still significantly above the control levels. Since *SNORD116* in PWS is completely silenced in PBMCs on maternal alleles, one would expect to see mat idic15 to have *SNORD116* expressed at normal levels (as one of the four alleles would be paternal and expressing *SNORD116* as in controls). The observed elevated levels, however, suggest that *SNORD116* on the paternal allele is being upregulated in PBMCs, though the mechanism is yet to be characterised. These findings should be confirmed in a larger independent cohort, as elevated levels of *SNORD116* mRNA from the paternal allele may contribute to mat idic15 pathology.

Moreover, in a study describing methylation-sensitive high-resolution melting-curve analysis on genomic DNA of post-mortem human brain tissues obtained from 8 Dup15q syndrome, 10 idiopathic autism and 21 typical control individuals^34^, PWS-IC methylation, and *UBE3A* transcript and protein levels were higher in Dup15q than in control or autism samples. Methylation of the PWS-IC region showed a positive correlation with *UBE3A* and *GABRB3* levels, but a negative correlation with *SNRPN* levels. This led the authors to suggest that gene expression within 15q11–q13 is not completely based on copy number changes, but it can also be influenced by epigenetic mechanisms in the brain.

### Genotype–phenotype relationships

In PWS (regardless of the molecular class) *UBE3A* mRNA levels were not significantly different compared to controls, while *SNORD16* expression was completely silenced. This was consistent with the real-time PCR results from an earlier study of *SNORD116* (referred to as *SNRPN* exons 57–58)^16^ that used exactly the same primers and probe as in this study. The previous study tested blood of two PWS participants, and also did not detect any *SNORD116* mRNA levels by real-time PCR. For quantitative analysis in AS and controls, however, it is not possible to accurately compare the results between the studies^16^ because: (i) the previous study reported quantitative real-time PCR results as a ratio of *SNORD116* over *UBE3A* mRNA, rather than normalising expression of each of the target genes to a set of stably expressed internal control genes; (ii) the number of participants was too low to perform statistical analysis as part of intergroup comparisons.

To examine potential clinical and biological significance of the detected variation in *UBE3A* and *SNORD116* mRNA levels in PBMCs, genotype–phenotype relationships were examined. Both *UBE3A* and *SNORD116* mRNA levels in PBMCs were significantly correlated with all age equivalent scores on the MSEL and the RRB CSS in the deletion AS group; higher gene expression levels were associated with better developmental functioning and less repetitive and restricted behaviours in this subtype. In the PWS deletion group, higher *UBE3A* mRNA levels were associated with lower SA CSS scores. In contrast, for the PWS non-deletion subtype, increased *UBE3A* mRNA levels were significantly associated with lower PIQ scores and increased SA CSS, reflecting poorer visuo-spatial and social communication skills, respectively. No significant associations were observed between *UBE3A* and ADOS scores in the Dup15q group. This may be attributed to the highly heterogenous molecular and clinical profiles of individuals with Dup15q and a significantly smaller sample size than the AS and PWS groups with available ADOS-2 data. Nonetheless, all Dup15q participants who completed the ADOS-2 (*n* = 8) met the cut-off for ‘Autism’ indicating a high level of autism symptoms

Overexpression of *UBE3A* has been linked to severity in Dup15q, where the increased number of maternal alleles is thought to be the primary driver of Dup15q pathology^34^. Similarly, PWS individuals with the UPD subtype are also thought to be ‘at a greater risk’ for ASD due to overexpression of *UBE3A*^35^. The findings provide support to this theory, however, it is possible that overexpression of *UBE3A* is more specifically related to the social communication deficits associated with ASD rather than RRBs. Several of the findings in this study support this notion: (i) higher *UBE3A* levels were associated with lower SA deficits in the PWS deletion subtype, which clustered at the bottom of the PWS group on *UBE3A* levels; (ii) in those with the non-deletion subtype higher *UBE3A* levels were associated with more SA difficulties; (iii) RRBs did not significantly differ across the three clinical groups, despite differences in *UBE3A* expression. Moreover, in ASD and other psycho-pathologies, such as schizotypy, gain of function *UBE3A* mutations have been suggested to be linked to phenotype severity^36–38^. It should be noted that ADOS scores in individuals with PWS may also be capturing other psychiatric difficulties including mood disorders and schizotypy traits, which are also linked to social communication deficits^39^. There is now a large amount of evidence supporting *UBE3A* dosage effects on schizotypy in the general population^38^ as well as those with PWS and Dup15q.

The associations between *UBE3A* and *SNORD116* mRNA levels and brain related processes, including developmental functioning and autism features, are consistent with the patho-mechanism proposed in a recent study examining transcriptomic signatures in the hypothalamus from post-mortem brain tissues in PWS^40^. This previous study demonstrated that genes associated with inflammatory response (including TNF-alpha and Il1-beta) were upregulated in PWS hypothalamus^40^. Together with the genotype-phenotype relationships reported in this study using PBMCs, this suggests that the immune response regulated by microglial cells (comprising almost half of all cells in the brain) may play a broader role in chromosome 15 imprinting disorder pathology. Abnormal expression of *UBE3A* in AS leucocytes, where *UBE3A* is not imprinted, may also have implications for clinical trials targeting *UBE3A* re-activation in the brain^41^. Specifically, *UBE3A* expression and regulation in other tissues may need to be monitored for non-specific impacts of the intervention on pathways regulated by *UBE3A* in non-neuronal cell types. Moreover, future studies should explore *UBE3A* expression in microglial cells in post-mortem human tissues of individuals affected with different chromosome 15 imprinting disorders, to confirm or refute these proposed patho-mechanisms.

### Strengths and limitations

One of the strengths of this study is the utility of ddPCR to accurately measure differences in gene expression of 30% or less—a feat not possible with most other methods including real-time quantitative PCR and RNA-seq^33^. Another strength is the characterisation of the most stably expressed internal control genes (*SDHA*, *ATP5B* and *EIF4A2*) using the geNorm approach in PBMCs of PWS and control groups^30^. This approach to select stably expressed reference genes for quantitative real-time PCR^30^ and ddPCR^42^ based studies has been widely recommended in other settings to ensure that the variation identified is not due to changes in genes used for target gene normalisation. Yet, previous studies for locus specific gene expression analysis in the chromosome 15 imprinting disorders in peripheral tissues and/or neurons, mostly utilised expression of *β*-actin^16^, *β*-2 microglobulin^15^ or GAPDH^32^ as a single internal control gene. Of note is that *β*-actin and *β*-2 microglobulin are involved in cell migration, which may be affected in chromosome 15 imprinting disorders^43^.

A limitation of the current study is the relatively small sample sizes for each of the subtypes in each condition as compared to similar studies performed in other rare disorders such as fragile X syndrome (FXS)^44^. Given these are rare genetic syndromes, obtaining large sample sizes can be difficult for each subtype. Nonetheless, this is one of the largest studies to date that has examined the utility of gene expression in peripheral tissues as biomarkers for brain related phenotypes in chromosome 15 imprinting disorders. Another limitation is that cellular and secreted protein product of *UBE3A* was not examined in this study. This is an important direction for future studies to confirm functional significance of changes in *UBE3A* mRNA levels and relationships with clinical phenotypes reported here.

While the use of peripheral tissues may not be entirely reflective of what is happening in the brain, the primary advantage of examining peripheral tissues is that the collection is less invasive than other tissues, such as cerebral spinal fluid (CSF), and will therefore likely increase sample sizes which is important for recruitment of individuals with rare genetic disorders. Evidence from other neurodevelopmental disorders, including FXS, has demonstrated significant relationships between promoter methylation, mRNA and protein and gene expression in peripheral tissues (e.g., blood and buccal epithelial cells) and the severity of the clinical phenotype, including intellectual functioning and autism features^22,44^. Nonetheless, future research exploring relationships between peripheral tissue biomarkers and other tissues such as CSF and brain tissue, where available, will further confirm the utility of peripheral biomarkers in understanding the molecular underpinnings of these rare genetic syndromes.

## Conclusion

In conclusion this study has demonstrated the utility of ddPCR for accurate quantification of gene expression in PBMCs in chromosome 15 imprinting disorders and characterisation of a stably expressed set of internal control genes. This ensured that variation reflected in the target genes analysed is not attributed to the variability in expression of the internal control gene/s used. *UBE3A* mRNA levels in PBMCs were shown to be significantly correlated with brain related phenotypes in both the PWS and AS cohorts. Specifically, increased *UBE3A* mRNA levels correlated with increased autism features in PWS and receptive language skills in AS, suggesting the utility of gene expression analysis in peripheral tissues as non-invasive biomarkers for future studies. Future research exploring clinical and biological significance of the relationships between *UBE3A* and *SNORD116* transcription in human peripheral and post-mortem brain tissues in PWS, AS and Dup15q patients are warranted to further explain the findings and the underlying mechanisms proposed in this study.

## Supplementary information

Supplemental Tables

Supplemental Figure legends

Supplemental Figure 1

Supplemental Figure 2
