# Supplementary material for: Relationships between UBE3A and SNORD116 expression and features of autism in chromosome 15 imprinting disorders
Source: Transl Psychiatry. 2020 Oct 29;10:362. doi: 10.1038/s41398-020-01034-7 (PMC7595031; doi:10.1038/s41398-020-01034-7)
Supplement: Supplementary file 1 — Supplemental Tables [file 41398_2020_1034_MOESM1_ESM.docx]

**Supplemental Table Legends**

**Supplemental Table S1:** **Genetic molecular classes for the PWS and AS groups.** *n* = sample size; PWS = Prader-Willi syndrome; AS = Angelman syndrome; Dup15q = Chromosome 15q Duplication; UPD = uniparental disomy; ICD = imprinting centre defect; Idic15 = Isodicentric 15q; Int15 = Interstitial 15q duplication

**Table S2**: **Summary statistics for intellectual functioning measures in the three groups.** N = sample size; Md = median; IQR = Interquartile range; VIQ= verbal IQ; PIQ = performance IQ; FSIQ = full scale IQ

**Table S3 Comparison of *UBE3A* and *SNORD116* expression between deletion and non-deletion groups (AS and PWS).** N = sample size; Md = median; IQR = Interquartile range; p = p-value*.*

**Table S4**: **Relationship between *UBE3A* and *SNORD116* expression and developmental functioning and autism features (AS cohort).** *β*  = estimated regression coefficient; s.e = standard error; p-value = *p*; CI = confidence interval; ^a^adjusted for age; *p-values remained <0.05 after Bonferroni correction.

**Table S5**: **Relationship between *UBE3A* expression and intellectual functioning and autism features (PWS cohort).** *β*  = estimated regression coefficient; s.e = standard error; p-value = *p*; CI = confidence interval; ^a^adjusted for age; *p-values remained <0.05 after Bonferroni correction.

**Table S6**: **Relationship between *UBE3A* and *SNORD116* expression and autism features (Dup15q cohort).** *β*  = estimated regression coefficient; s.e = standard error; p-value = *p*; CI = confidence interval; ^a^adjusted for age.

**Supplemental Table S1:** Genetic molecular classes for the PWS and AS groups

|  | **PWS**  (*n* = 27) | **AS**  (*n* = 21) | **Dup15q**  (*n* = 10) |
| --- | --- | --- | --- |
| Deletion | 37.0% | 47.6% | - |
| UPD | 59.1% | 14.3% | - |
| ICD | 11.1% | 4.8% | - |
| *UBE3A* mutation | - | 28.6% | - |
| Methylation Mosaic | - | 4.8% | - |
| Idic15q | - | - | 60.0% |
| Int dup15q | - | - | 30.0% |
| Tricentric 15q | - | - | 10.0% |

*n* = sample size; PWS = Prader-Willi syndrome; AS = Angelman syndrome; Dup15q = Chromosome 15q Duplication; UPD = uniparental disomy; ICD = imprinting centre defect; Idic15 = Isodicentric 15q; Int15 = Interstitial 15q duplication

**Table S2**: Summary statistics for intellectual functioning measures in the three groups

|  | **PWS** | | |  | **AS** | | |  | **Dup15q** | | |  |
| --- | --- | --- | --- | --- | --- | --- | --- | --- | --- | --- | --- | --- |
|  | N | Md | IQR |  | N | Md | IQR |  | N | Md | IQR |  |
| VIQ | 27 | 67.0 | 17.0 |  | - | - | - |  | 3 | 75.0 | - |  |
| PIQ | 27 | 65.0 | 18.0 |  | - | - | - |  | 3 | 57.0 | - |  |
| FSIQ | 27 | 65.0 | 21.0 |  | - | - | - |  | 3 | 67.0 | - |  |
| Visual Reception age equivalent (mths) | - | - | - |  | 20 | 18.5 | 13.0 |  | 6 | 11.5 | 4.0 |  |
| Fine Motor age equivalent (mths) | - | - | - |  | 20 | 18.0 | 12.5 |  | 6 | 14.5 | 2.0 |  |
| Receptive Language age equivalent (mths) | - | - | - |  | 20 | 25.0 | 14.5 |  | 6 | 11.0 | 7.0 |  |
| Expressive Language age equivalent (mths) | - | - | - |  | 20 | 12.0 | 9.0 |  | 6 | 11.0 | 6.0 |  |

N = sample size; Md = median; IQR = Interquartile range; VIQ= verbal IQ; PIQ = performance IQ; FSIQ = full scale IQ

**Table S3** Comparison of *UBE3A* and *SNORD116* expression between deletion and non-deletion groups (AS and PWS).

|  | **Deletion** | | |  | **Non-deletion** | | | |
| --- | --- | --- | --- | --- | --- | --- | --- | --- |
|  | N | Md | IQR |  | N | Md | IQR | p |
| **AS** |  |  |  |  |  |  |  |  |
| *UBE3A* | 10 | 0.085 | 0.020 |  | 11 | 0.184 | 0.040 | **0.002** |
| *SNORD116* | 10 | 0.032 | 0.011 |  | 11 | 0.041 | 0.041 | 0.197 |
| **PWS** |  |  |  |  |  |  |  |  |
| *UBE3A* | 10 | 0.086 | 0.021 |  | 17 | 0.200 | 0.037 | **<0.001** |

N = sample size; Md = median; IQR = Interquartile range; p = p-value*.*

**Table S4**: Relationship between *UBE3A* and *SNORD116* expression and developmental functioning and autism features (AS cohort).

| Outcome | *n* | *β* | s.e | *p* | 95%CI |
| --- | --- | --- | --- | --- | --- |
| ***UBE3A* - predictor** |  |  |  |  |  |
| Visual Reception | 20 | 95.0 | 41.4 | **0.022** | (13.9, 176.0) |
| Fine Motor^a^ | 20 | 82.2 | 33.8 | **0.015** | (15.9, 159.0) |
| Receptive Language | 20 | 134.0 | 37.0 | <**0.001*** | (61.4, 206.0) |
| Expressive Language^a^ | 20 | 69.7 | 9.6 | <**0.001*** | (50.9, 88.4) |
| ADOS CSS | 20 | -6.5 | 7.1 | 0.357 | (-20.4, 7.4) |
| SA CSS | 20 | -11.2 | 8.8 | 0.199 | (-28.4, 5.9) |
| RRB CSS | 20 | -5.4 | 5.1 | 0.285 | (-15.3, 3.5) |
| ***SNORD116* - predictor** |  |  |  |  |  |
| Visual Reception | 20 | 43.4 | 83.3 | 0.602 | (-120.0, 207.0) |
| Fine Motor^a^ | 20 | 53.9 | 68.9 | 0.434 | (-81.1, 189.0) |
| Receptive Language | 20 | 95.2 | 102.0 | 0.351 | (-105.0, 295.0) |
| Expressive Language^a^ | 20 | 91.3 | 48.8 | 0.061 | (-4.4, 187.0) |
| ADOS CSS | 20 | -6.8 | 11.7 | 0.561 | (-29.7, 16.1) |
| SA CSS | 20 | -15.2 | 13.4 | 0.258 | (-41.5, 11.1) |
| RRB CSS | 20 | -10.3 | 8.8 | 0.241 | (-27.6, 6.9) |

*β*  = estimated regression coefficient; s.e = standard error; p-value = *p*; CI = confidence interval; ^a^adjusted for age; *p-values remained <0.05 after Bonferroni correction.

**Table S5**: Relationship between *UBE3A* expression and intellectual functioning and autism features (PWS cohort)

|  | *n* | *β* | s.e | p | 95% CI |
| --- | --- | --- | --- | --- | --- |
| ***UBE3A* - predictor** | | | | | |
| VIQ | 27 | -49.7 | 41.6 | 0.233 | (-131, 31.9) |
| PIQ | 27 | -121 | 27.7 | **<0.001*** | (-175, -66.5) |
| FSIQ | 27 | -70.6 | 39.1 | 0.071 | (-147, 6.07) |
| ADOS CSS^a^ | 25 | 16.7 | 6.59 | **0.011*** | (3.84, 29.7) |
| SA CSS^a^ | 25 | 18.1 | 5.66 | **0.004*** | (6.33, 29.9) |
| RRB CSS | 25 | 6.41 | 9.21 | 0.486 | (-11.6, 24.5) |

*β*  = estimated regression coefficient; s.e = standard error; p-value = *p*; CI = confidence interval; ^a^adjusted for age; *p-values remained <0.05 after Bonferroni correction.

**Table S6**: Relationship between *UBE3A* and *SNORD116* expression and autism features (Dup15q cohort).

|  | *n* | *β* | s.e | *p* | 95% CI |
| --- | --- | --- | --- | --- | --- |
| ***UBE3A* – predictor** | | | | | |
| ADOS CSS^a^ | 8 | -2.52 | 2.92 | 0.387 | (-8.24, 3.19) |
| SA CSS^a^ | 8 | -3.55 | 3.75 | 0.345 | (-10.9, 3.81) |
| RRB CSS | 8 | 10.5 | 9.16 | 0.250 | (-7.42, 28.5) |
| ***SNORD116* – predictor** | | | | | |
| ADOS CSS^a^ | 8 | 21.1 | 16.7 | 0.207 | (-11.7, 53.9) |
| SA CSS^a^ | 8 | 53.6 | 27.6 | 0.109 | (-17.2, 124) |
| RRB CSS | 8 | 23.0 | 106 | 0.829 | (-185, 231) |

*β*  = estimated regression coefficient; s.e = standard error; p-value = *p*; CI = confidence interval; ^a^adjusted for age.
