# Supplementary material for: Relationships between UBE3A and SNORD116 expression and features of autism in chromosome 15 imprinting disorders
Source: Transl Psychiatry. 2020 Oct 29;10:362. doi: 10.1038/s41398-020-01034-7 (PMC7595031; doi:10.1038/s41398-020-01034-7)
Supplement: Supplementary file 2 — Supplemental Figure legends [file 41398_2020_1034_MOESM2_ESM.docx]

**Supplemental Figure 1. Establishing positive call threshold and dynamic linear range (DLR) for singleplex droplet digital PCR internal control gene assays on serially diluted RNA reference sample, with RNA input ranging between 170.4 and 1.33 ng. (A-H)** The geNorm gene panel of internal control genes *SDHA, ATP5B, EIF4A2, RPL13A, GAPDH, TOP1* and *YWHAZ***.** Note: Positive amplitude thresholds were established on the ddPCR 2-D plots using no template controls (no RNA input – 1^st^ column of each 2-D plot included in every run) at the Amplitude of the droplet/s with the highest amplitude unit value. *GAPDH* was one exception to this rule, where relationship between observed positive droplet number (X-axis) and expected RNA input was also examined at the second positive threshold (5999 Amplitude units) **(E),** which was below the droplet cluster that comprised the majority positive droplets not present in the no template control. **Note:** The order of samples in the columns for each 2-D plot from (left to right), is consistent the order of rows (top to bottom) in each adjacent table. Similarly, for *RPL13A* relationship between observed positive droplet number (X-axis) and expected RNA input was also examined at the positive threshold (2784 Amplitude units) **(G),** which was below the droplet cluster that comprised the majority positive droplets not present in the no template control. Dynamic linear range was defined based on presence of a linear relationship between mean (labelled as ST1-ST6) observed positive droplet number (Y-axis) [from 2 technical replicates (labelled as gene name followed by .1 or .2)] and expected RNA input in ng (X-axis). Maximum and minimum values for each DLR are highlighted by red arrows next to each table for every gene included.

**Supplemental Figure 2. Establishing positive call threshold and dynamic linear range (DLR) for a douplex droplet digital PCR assay targeting *UBE3A* and *SNORD116* on serially diluted RNA reference sample, with RNA input ranging between 170.4 and 1.33 ng. (A)** *UBE3A* mRNA assessed using positive 2-D amplification threshold of 2,735 units; **(B)** *SNORD116* using positive 2-D amplification threshold of 1,452 units. **Note:** The order of samples in the columns for each 2-D plot from (left to right), is consistent the order of rows (top to bottom) in each adjacent table. Established dynamic linear range for each assay was based on presence of a linear relationship between was defined based relationship between the observed positive droplet number (Y-axis) and expected RNA input in ng. Maximum and minimum values (X-axis) for each DLR are highlighted by red arrows next to each table for every gene included.
