# Supplementary material for: Relationships between UBE3A and SNORD116 expression and features of autism in chromosome 15 imprinting disorders
Source: Transl Psychiatry. 2020 Oct 29;10:362. doi: 10.1038/s41398-020-01034-7 (PMC7595031; doi:10.1038/s41398-020-01034-7)

Supplemental Figure 1.

A.

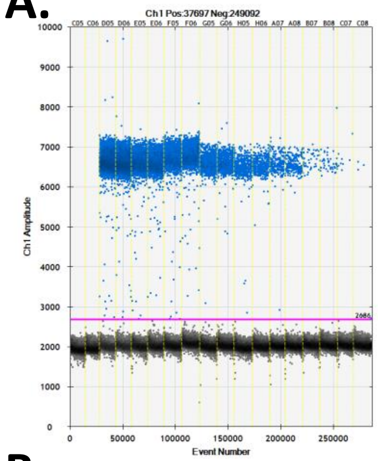

RNA input amount(ng)	ATP5B.1	ATP5B.2	Mean
0	0	0	0
170.4	22580	22860	22720
85.2	8320	8180	8250
42.6	4280	4640	4460
21.3	1866	1896	1881
10.65	660	624	642
5.325	338	300	319
2.6625	58	60	59
1.33125	13	6.6	9.8

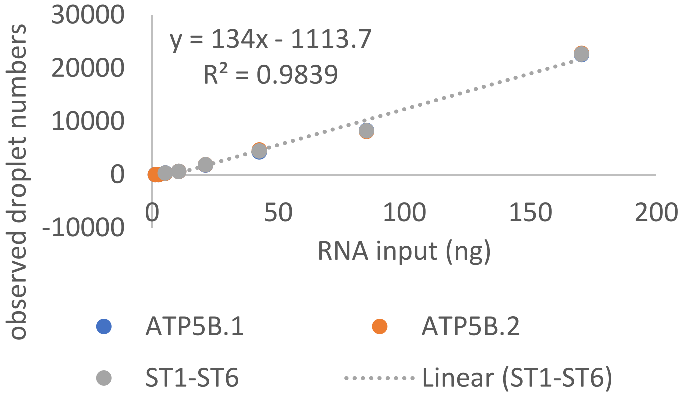

B.

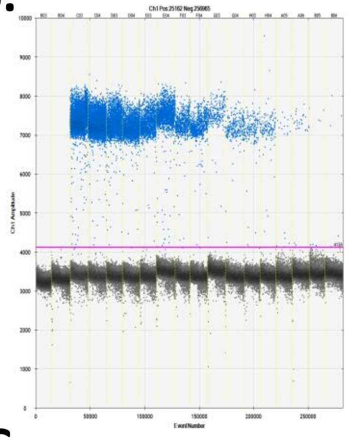

RNA input amount(ng)	SDHA.1	SDHA.2	Mean
0	0	0	0
170.4	12980	12060	12520
85.2	5100	4940	5020
42.6	2514	2600	2557
21.3	1150	1144	1147
10.65	340	360	350
5.325	252	218	235
2.6625	30	46	38
1.33125	10	7.2	8.6

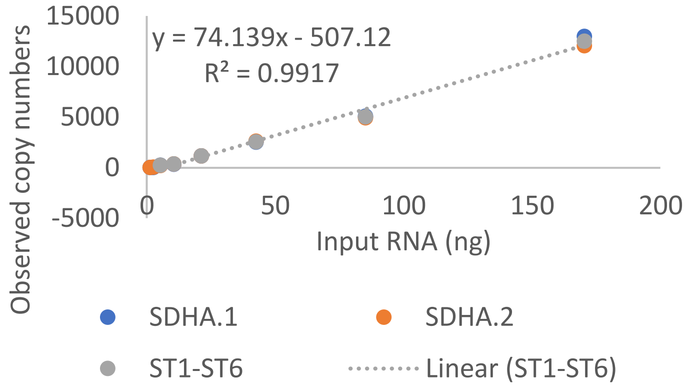

C.

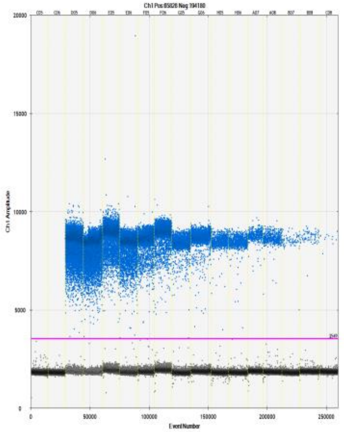

RNA input amount(ng)	EIFYA4.1	EIFYA4.2	Mean
0	0	0	0
170.4	54760	52440	53600
85.2	21600	20680	21140
42.6	11120	11480	11300
21.3	4640	4580	4610
10.65	1508	1400	1454
5.325	798	858	828
2.6625	120	118	119
1.33125	19.6	12	15.8

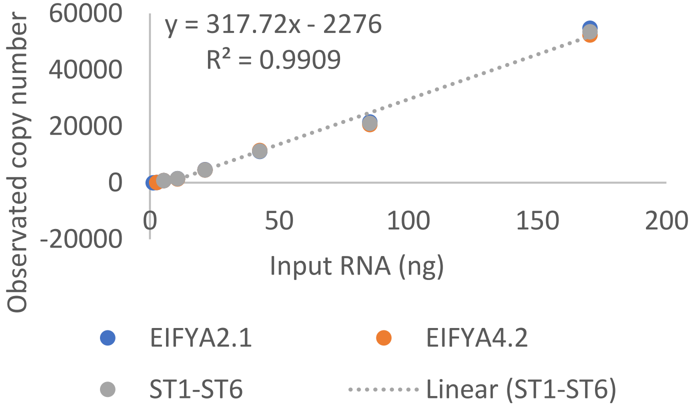

Supplemental Figure 1.

D.

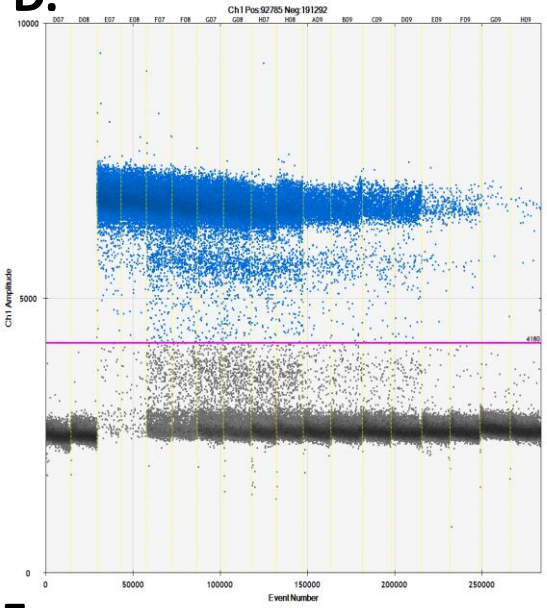

RNA input amount(ng)	GAPDH.1	GAPDH.2	Mean
0	0	0	0
170.4	117800	122600	120200
85.2	49200	49800	49500
42.6	24300	23620	23960
21.3	11120	10980	11050
10.65	3780	3920	3850
5.325	1900	1706	1803
2.6625	294	240	267
1.33125	36	36	36

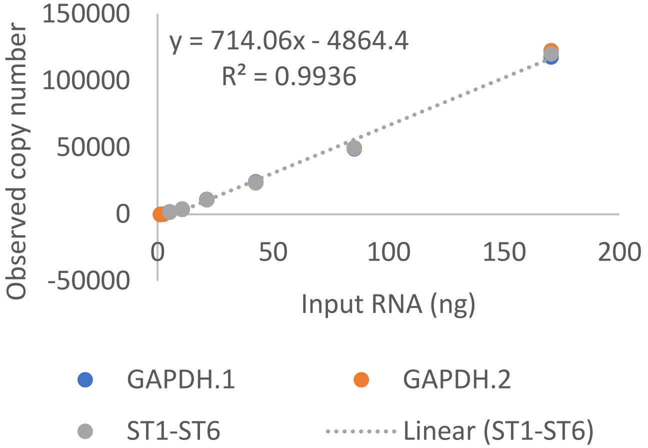

E.

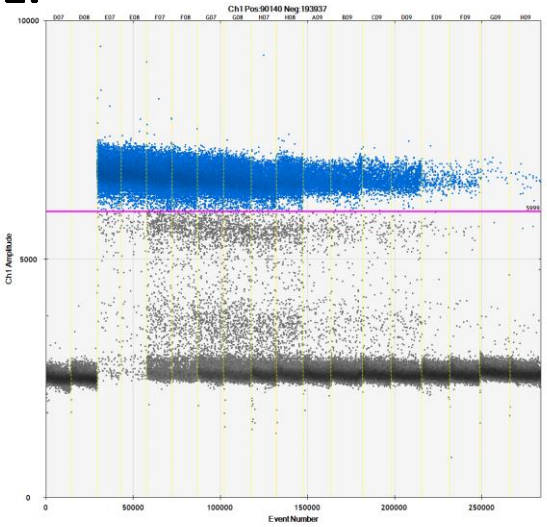

RNA input amount(ng)	GAPDH.1	GAPDH.2	Mean
0	0	0	0
170.4	104200	110000	107100
85.2	44920	45500	45210
42.6	22580	22020	22300
21.3	10360	10320	10340
10.65	3600	3740	3670
5.325	1780	1604	1692
2.6625	268	226	247
1.33125	34	32	33

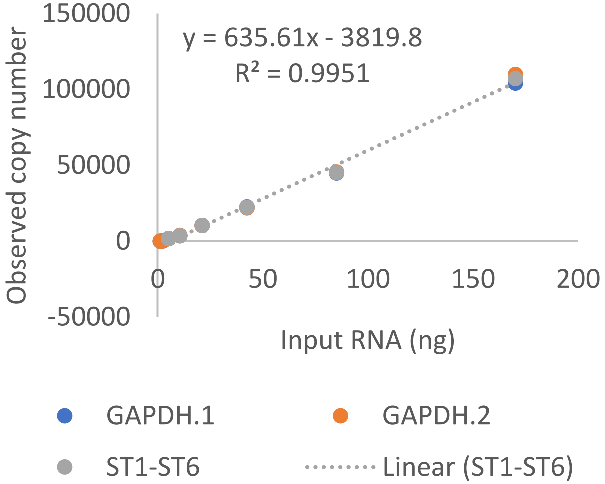

Supplemental Figure 1.

F.

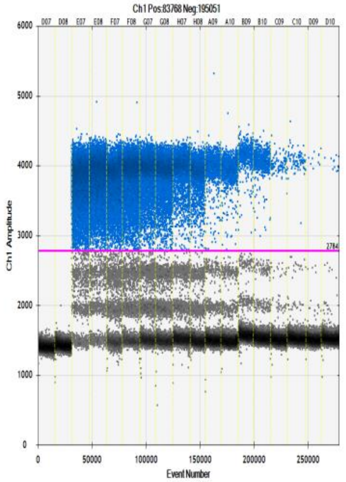

RNA input amount(ng)	RPL13A.1	RPL13A.2	RPL13A Mean
0	0	0	0
170.4	67000	67800	67400
85.2	33760	35120	34440
42.6	18480	17840	18160
21.3	7980	7680	7830
10.65	2468	2462	2465
5.325	1134	1184	1159
2.6625	142	126	134
1.33125	16.6	15.8	16.2

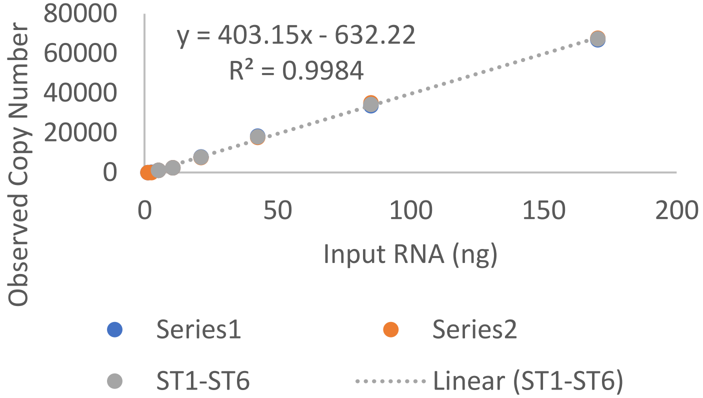

G.

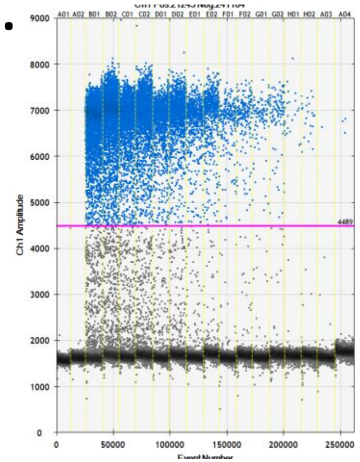

RNA input amount(ng)	TOP1.1	TOP1.2	Mean
0	0	0	0
170.4	10780	11660	11220
85.2	4700	4580	4640
42.6	2362	2360	2361
21.3	934	1014	974
10.65	302	308	305
5.325	158	182	170
2.6625	30	36	33
1.33125	1.4	4.2	2.8

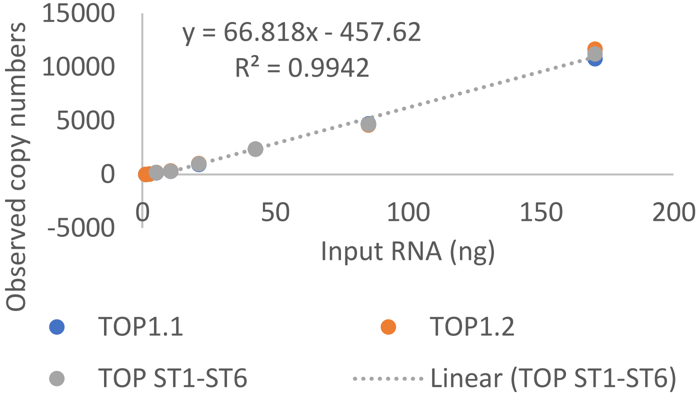

H.

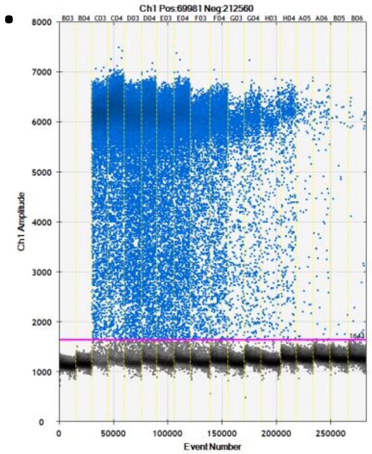

RNA input amount(ng)	YWHAZ.1	YWHAZ.2	Mean
0	0	0	0
170.4	60000	63400	61700
85.2	24700	25800	25250
42.6	12400	12820	12610
21.3	5000	4860	4930
10.65	1364	1590	1477
5.325	790	862	826
2.6625	150	134	142
1.33125	25.6	34	29.8

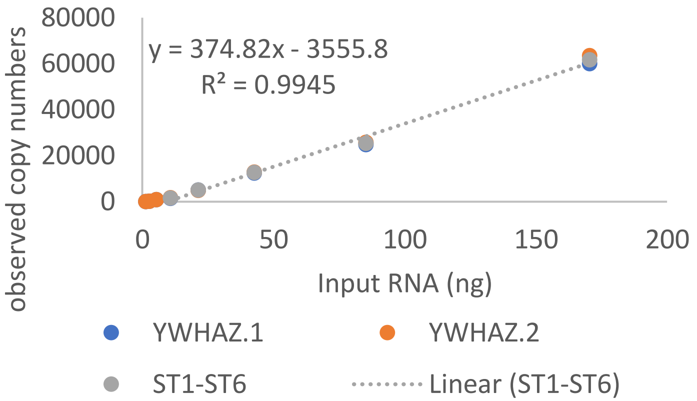

Supplement: Supplementary file 3 — Supplemental Figure 1 [file 41398_2020_1034_MOESM3_ESM.pdf]
