# Supplementary material for: Relationships between UBE3A and SNORD116 expression and features of autism in chromosome 15 imprinting disorders
Source: Transl Psychiatry. 2020 Oct 29;10:362. doi: 10.1038/s41398-020-01034-7 (PMC7595031; doi:10.1038/s41398-020-01034-7)
Supplement: Supplementary file 4 — Supplemental Figure 2 [file 41398_2020_1034_MOESM4_ESM.pptx]

## Slide 1
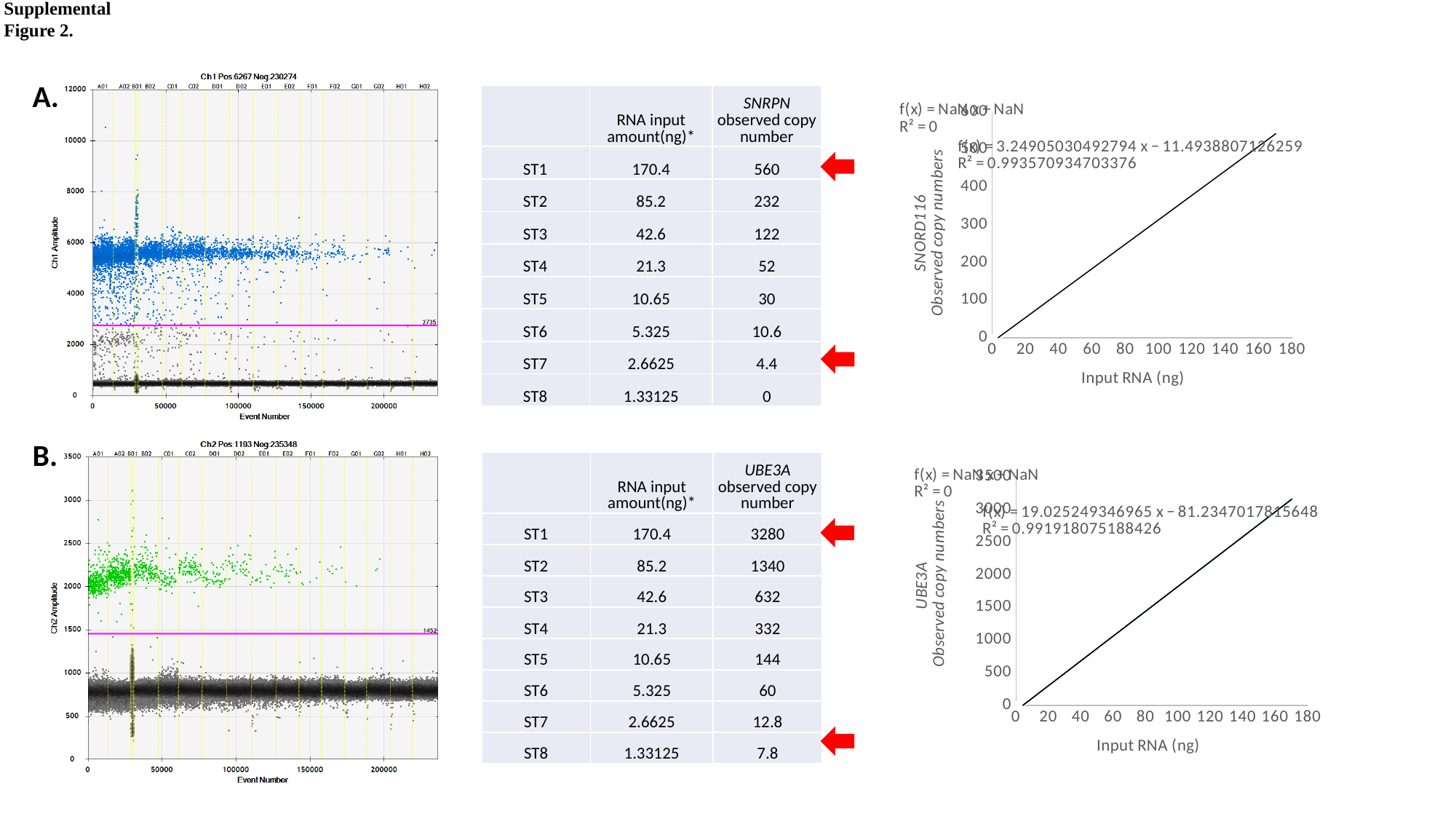

Supplemental
Figure 2.
A.
| | RNA input amount(ng)\* | SNRPN observed copy number |
| --- | --- | --- |
| ST1 | 170.4 | 560 |
| ST2 | 85.2 | 232 |
| ST3 | 42.6 | 122 |
| ST4 | 21.3 | 52 |
| ST5 | 10.65 | 30 |
| ST6 | 5.325 | 10.6 |
| ST7 | 2.6625 | 4.4 |
| ST8 | 1.33125 | 0 |
### Chart
| Category | SNRPN | |
|---|---|---|
B.
| | RNA input amount(ng)\* | UBE3A observed copy number |
| --- | --- | --- |
| ST1 | 170.4 | 3280 |
| ST2 | 85.2 | 1340 |
| ST3 | 42.6 | 632 |
| ST4 | 21.3 | 332 |
| ST5 | 10.65 | 144 |
| ST6 | 5.325 | 60 |
| ST7 | 2.6625 | 12.8 |
| ST8 | 1.33125 | 7.8 |
### Chart
| Category | UBE3A | |
|---|---|---|
